# "The Hospital" Medical Book Supplement.—No. XI

**Published:** 1908-10-17

**Authors:** 


### "THE HOSPITAL"
### Medical Book Supplement.—No. XI.

CONTENTS.

**Medicine**—

Diseases of the Spinal Cord ... 71

Common Affections of the Liver ... 71

The Ophthalmic and Cutaneous Diagnosis of Tuberculosis ... 71

**Surgery**—

Injuries of Nerves and their Treatment ... 72

Hernia: Its Cause and Treatment ... 72

Cancer ... 72

Manual of Ophthalmic Medicine and Surgery ... 72

**Public Health and Hygiene**—

The National Physique ... 73

The Feeding of the Infant ... 73

**Massage**—

Le Ventre: Etude Anatomique et Clinique de la Cavité Abdominale au Point de vue du Massage ... 73

**Embryology**—

Quain's Anatomy ... 73

**Miscellaneous Items**—

The Theory of Ions: A Consideration of its Place in Biology and Therapeutics... 74

Britain's Blot ... 74

The Black Death ... 74

The Solar System ... 74

On Means for the Prolongation of Life ... 74

Health Resorts of Europe ... 74

### MEDICINE.

Diseases of the Spinal Cord. By R. T. Williamson, M.D., F.R.C.P. (Oxford Medical Publications. Henry Frowde and Hodder and Stoughton. Pp. 432. 183 figures. Price 15s. net.)

This book is an amplification of a course of lectures delivered by the author during the past fifteen years at the Manchester Medical School, on which it reflects credit. It presents in clear language a reliable account of our present views on diseases of the spinal cord. One of its most conspicuous merits is that whilst taking full cognisance of contemporary neurological literature, the writer succeeds in preserving a personal atmosphere, a refreshing thing in dealing with subjects on which too many authors are mere wielders of scissors and paste. It is impossible in a brief review to give an adequate account of the material so assiduously collected. After introductory chapters on the structure of the spinal cord and on its general pathological histology, the writer discusses the functions of the spinal cord and gives an account of the symptoms of spinal diseases; this latter chapter, owing to its relative brevity, is hardly up to the standard of the rest of the book. Electrical examination, skiagraphy and lumbar puncture are placed together and discussed in a special chapter. Then follow interesting chapters on the diagnosis and localisation of the various spinal-cord diseases, with an appendix on the methods of pathological examination. The references to other authorities are partly included in the text, partly grouped in lists at the end of each chapter; from the reader's point of view it would have been better to adopt one or other of these arrangements, not both. As a whole, however, the book is an excellent one and will be most valuable both to the senior student and to the practitioner.

Common Affections of the Liver.—By W. Hale White, M.D., F.R.C.P., Senior Physician to and Lecturer on Medicine at Guy's Hospital. (London: James Nisbet and Co. 1908. Pp. viii. + 302. Price 4s. 6d. net.)

This is not a text-book, but rather a series of clinical lectures upon the liver. It represents bedside teaching, and as such it possesses that personal charm which is unfortunately lacking in so many modern works of multiple authorship. Treatment is entered into in greater detail than is usually the case in clinical lectures, and the advice given is most practical and to the point. It might be expected that "biliousness," "torpidity of the liver," and so forth, would find a large place in a work bearing the title "Common Affections of the Liver." Readers who expect this will perhaps be disappointed to find them dealt with in a single page, and excluded upon the ground that they have probably little or nothing to do with the liver at all. The author's remarks upon the subject are crisp and concise, but we should very much like to have been given a full clinical lecture upon "biliousness." Although biliousness is thus excluded from the conditions dealt with at any length, the reader will find admirable chapters upon such subjects as jaundice, displaced liver, diseases of the liver vessels, congestion of the liver, suppuration in and around the liver, cirrhosis of the liver, perihepatitis, syphilis of the liver, malignant disease of the liver, tuberculous, lardaceous, actinomycotic, and hydatid diseases of the liver, acute yellow atrophy, and icterus neonatorum. The book is of convenient size, and the type is clear and large; the volume is light to hold, pleasant to read, and, at the same time, most instructive.

The Ophthalmic and Cutaneous Diagnosis of Tuberculosis. By Dr. Alfred Wolff-Eisner. Translated from the German by Bernard I. Robert. Large 8vo. Pp. viii + 207; 2 coloured plates; 15 figures. (London: John Bale, Sons, and Danielsson, Ltd.

This book gives an interesting and fairly clear account of the origin, technique, applicability, usefulness, and comparative value of the tuberculo-cutaneous and of the tuberculo-ophthalmic reactions for the detection of tuberculous lesions. It is a pity that there should be any soreness of feeling between Calmette on the one hand, and Wolff-Eisner on the other, as to which of the two was the first to discover what is now generally known as "Calmette's reaction," but which, it seems, was discovered by Wolff-Eisner; and one cannot help feeling that there is an uncomfortable strain of "I was really the first to discover this" all through the book. This, together with the fact that there is no index, is a serious objection to the volume; but the descriptions given by the author, both of the methods themselves and of the results that may be obtained by their use in all kinds of conditions, render the book one which will be of considerable assistance to medical practitioners. It is probable that in time certain objections will be found to both the cutaneous and the conjunctival tuberculo-reactions, and limitations to their value; such objections and limitations are scarcely to be looked for in the book of an enthusiast; but notwithstanding that, the author here gives a very fair summary of what has been found out up to the present. The translator leaves the style distinctly German in many places.

### SURGERY.

Injuries of Nerves and Their Treatment. By James Sherren, F.R.C.S.Eng., Assistant Surgeon London Hospital. (London: James Nisbet and Company. Price 5s. net).

With much that appears in this volume we have been made familiar by Mr. Sherren's previous writings, and we are now afforded a review of the whole subject which cannot fail to be of the greatest interest to every surgeon. The results from the researches of the author and Dr. Head are briefly reviewed. We have still some doubts as to the anatomical entity of separate fibres for epicritic and protopathic sensation; it is a matter which seems hardly to admit of experimental proof, but be the dissociation of function anatomical or physiological there can be no doubt of the value of the findings to the clinician. The great advance in neurological diagnosis that has been brought about by the study of dissociation of sensation is nowhere better illustrated than by the ease with which we are now able to differentiate between lesions of the conus and cauda equina by considering whether the segmental sensory loss be of peripheral or cord type, though, of course, this is only true in the—fortunately commoner—event of the cauda lesion being incomplete. In speaking of the reaction of degeneration the author does well not to lay stress on the complicated phenomena of polar change with which it is unfortunately still the custom to torment the student. The experience of the author in facial-hypoglossal anastomosis must be happier than our own when he says that "in any case we may confidently predict great improvement to follow the operation." The thorny subject of peripheral regeneration is treated briefly; it has always appeared to us that the results of Betlu with continuous nerve loops give an almost incontrovertible proof of autogenous peripheral degeneration in the young mammal. We are far from sure that the early use of electrical treatment recommended is advisable. There is experimental evidence that a cut nerve degenerates far more rapidly when subjected to faradic stimulation than when left alone. It is probably better to defer electrical treatment till signs of regeneration have set in. The book is well illustrated by photographs of cases; an inspection of that on page 180 shows the well-marked effect of sympathetic palsy on the pilomotor muscles of the eyebrows, a clinical sign frequently overlooked. The work can be warmly recommended to both surgeons and neurologists, and constitutes a most valuable addition to our knowledge of the clinical pathology of the peripheral nervous system.

Hernia: Its Cause and Treatment. By R. W. Murray, F.R.C.S., Surgeon to the David Lewis Northern Hospital, Liverpool, late Surgeon to the Liverpool Infirmary for Children. (London: J. and A. Churchill, 7 Great Marlborough Street. 1908. Pp. 99.)

Since the introduction of antiseptic surgery the treatment of hernia by trusses has been largely superseded by operation for radical cure. In fact it is no exaggeration to say that a radical cure is nowadays performed in every case if there is no real contraindication to operation; in this way our knowledge of the state of affairs found in herniæ has become very exact. In this book Mr. Murray has some remarks to make on the causation and on the treatment of hernia. He is strongly inclined to the view that whether a hernia be "congenital" or "acquired," the peritoneal sac which forms its covering is present beforehand: in other words, the hernia descends into a potential sac already present, and rarely, if ever, pushes its peritoneal investment before it. In this sense all herniæ, except perhaps the direct variety, are congenital, and it is an accident which determines whether the sac is formed by the unobliterated processus vaginalis or by an accessory pouch of peritoneum unconnected with this. It follows that in the question of treatment the all-important factor is to remove the sac as completely as possible. As long as this is done the result will be equally good whatever method be adopted for the rest of the operation. The author advocates a method, first introduced by Lucas Championnière, in which the edges of the cut aponeurosis are made to overlap, and he has followed up a number of his cases to prove that the after results are satisfactory. Undoubtedly they are, but then so they are by any method as long as the sac is completely removed.

Cancer. By G. Sherman Bigg, F.R.C.S.(Edin.), M.R.C.S.(Eng.), L.S.A.; late Surgeon-Captain Army Medical Staff, etc. (London: Baillière, Tindall, and Cox. 1907. Crown 8vo. Pages vii. + 85. Price 3s. 6d. net.)

Many small publications, of a quasi-scientific character, upon cancer are cropping forth just now, and we have already expressed our views about the printing of immature and one-sided views upon the subject. There is nothing either new or striking in the volume before us, nor does it contain anything very helpful. The views expressed by the author have long ago been expressed by others, and a very great deal of research work has been, and is still being, done upon them by those who are far better acquainted with the subject than Mr. Bigg seems to be. One point in the volume that merits attention is its last paragraph but one: "Scientific research starts from near the finish, and not from the commencement, of the disease, so that the investigation is not only incomplete, but is inaccurate." It is quite clear from this that Mr. Bigg is unfamiliar with the immense amount of histological work done by such skilled authorities as Professor Ribbert. The theories that Mr. Bigg propounds are neither new nor his own; and although he says in his preface that "the right to an opinion is a universal privilege, but the expression of it becomes a duty when it is considered to be for the good and benefit of others," we think it a pity that Mr. Bigg felt that it was a duty to cumber the world with a wholly unnecessary publication.

---

Manual of Ophthalmic Medicine and Surgery. By W. H. Jessop, M.A., M.B., F.R.C.S., Senior Ophthalmic Surgeon to St. Bartholomew's Hospital, etc. (London: J. and A. Churchill, 1908. Second Edition. Pp. 531. Price 9s. 6d. net.)

The composition of a text-book on diseases of the eye is probably as difficult a task as any in medical literature; a text-book, that is to say, designed for students and practitioners. The subject is a vast and complicated one, and it must be condensed into a compass suitable to the needs of those who have but little time to devote to the subject. On the other hand, there must be included some account of every condition which may be met with in practice, and of its treatment, and there must be an attempt to explain two processes which most students find inexplicably difficult—retinoscopy and the examination of the fundus. It follows that anyone who starts out to write such a book must be both an experienced surgeon and a sympathetic teacher; we should imagine from the internal evidence of this book that Mr. Jessop is both. The critical eye notes two minor omissions, at least; but, taken as a whole, the book is excellent and well worthy of the popularity of which this re-issue is evidence.

### PUBLIC HEALTH AND HYGIENE.

The National Physique. By A. Stayt Dutton, L.R.C.P. Lond., M.R.C.S. Eng. (London: Baillière, Tindall, and Cox. 1908. Pp. xii. + 188. Price 5s. net.)

It would be difficult to guess from the title of this book precisely what sort of subject-matter it contains. It would even be difficult to say whether it is intended for the laity, or for the profession, or for both. It certainly comes as a surprise to find that it is practically a lay sermon upon the text "Anæmia." There is doubtless a deal of truth in what the author says, and we are far from pooh-poohing the work; but we feel that when he traces the cause of almost every ailment to anæmia he is going too far, just as we think that those do, too, who look upon uric acid as the source of most human illnesses. There is little doubt that work in towns and cities, in workshops and in factories, tends to diminish the richness of the blood in hæmoglobin. We agree that persons whose blood is below par are less able to resist disease than are those whose blood is in perfect order. We agree that in the best interests of the national physique each individual should live as far as possible such a life as will not tend to impoverish the blood. Fresh air and sunlight are amongst the best remedies for anæmia. We become weary, however, of the constant reiteration of the word "Anæmia." The title would have made the nature of the subject-matter clearer had it been "Anæmia, the Main Cause of Physical Deterioration and of Many Common Ailments."

The Feeding of the Infant. By J. W. Simpson, M.D., F.R.C.P.Edin., Assistant Physician Sick Children's Hospital, Edinburgh. (Edinburgh: James Thin. Pp. 80.)

Dr. Simpson has written a small and intelligible pamphlet in book form admirably suited for the instruction of mothers or nurses on the simple methods of diet and on some of the minor troubles connected therewith. Thirty-three pages are devoted to breast-feeding, two to weaning, and the rest to artificial feeding. The advice given is sound and practical. We do not countenance two-hourly feeds for as long as six weeks to two months, and think the intervals should be prolonged to three hours before the end of the fourth month. The scalding or boiling of milk is recommended under certain circumstances, but no reference is made to the necessity of rapidly cooling the milk and keeping it in the cold if it has to be kept for further use. We suggest to the author that on revision more precise tables for diet during the first month should be given, and that the gradual education of the child in the digestion of starch should be brought about by the introduction of barley-water into the milk mixtures during the third quarter of the first year of life. Of the authorities quoted Cautley deserves to have his name properly spelt. The book is quite useful, and with a little care could be made still more so without unduly enlarging its scope.

### MASSAGE.

Le Ventre : Etude Anatomique et Clinique de la Cavité Abdominale au Point de vue du Massage. Vol. II. : "L'Estomac et L'Intestin." Par Dr. F. Cauteu et Dr. M. Bourcart. (Paris : Félix Alcan. 1908. Pp. 335. 81 illustrations. Large 8vo.)

There is one point which is bound to limit the extent to which this work is read in Great Britain—namely that of language—it is written entirely in French. It is clear, however, that it should find a place in every medical or surgical library in this country. The views of the authors are no doubt biassed, as they are almost bound to be, in favour of the beneficial effects of massage in a great many different abdominal conditions; but the volume is not "massage" from end to end. There is a long chapter devoted entirely to the anatomy of the stomach and intestines, with their blood and nerve supplies, and a very great deal of trouble has been spent upon large and good illustrations which cannot but be useful both to anatomists and to surgeons. Another long chapter is devoted entirely to the physiology of digestion, including not only secretion, chemical action, and absorption; but also motility, a part of the subject which is apt to be left out in text-books of physiology. The authors devote another chapter to the effects of massage upon the physiology of digestion; they do not merely state beliefs; they have carried out extensive experiments, gastric juice analyses, and so forth, and they draw conclusions from these as to the way massage of the abdomen acts upon the activities of the underlying viscera. They give further experimental results to show the actual results of massage in cases of chronic constipation, biliousness, diarrhœa, hyperchlorhydria, nervous, cardiac, and anæmic dyspepsias, mucous colitis, enteroptosis, gastrectasis, the vomiting of pregnancy, the after-effects of sea-sickness, and so forth. Not only this, however, but they also give precise details of the methods of abdominal massage, illustrated by very clear full-page pictures of the different stages and processes. We think the publication steers clear of fads to a very considerable extent, and, although it has massage for its basis, we think it includes so much besides that it must be regarded as a scientific treatise containing points that interest the anatomist, the surgeon, the physiologist, the physician, the general practitioner, and the specialist in massage. We may add that the entire publication consists of three volumes, of which the above is the second.

### EMBRYOLOGY.

Quain's Anatomy. Vol I. Embryology. 11th Edition. Editors : E. A. Schäfer, LL.D., Sc.D., F.R.S.; Johnson Symington, M.D., F.R.S.; and T. H. Bryce, M.A., M.D. Pp. 275, with 300 illustrations. (London : Longmans, Green and Co. 1908. Price 10s. 6d. net.)

The recent advances in our knowledge of human embryology have necessitated very considerable additions and changes in this volume of "Quain's Anatomy," and the present edition has been re-written by Dr. Bryce and many new illustrations have been added. The first section on "General Embryology," which will of course be eagerly read, is perhaps the least satisfactory. It is questionable whether it would not be wiser to confine the attention to human embryology in a work which is essentially a treatise on human anatomy. The excursions into comparative embryology lead to considerable confusion, and it is not always clear whether reference is made to human embryos or to those of some other animals, *e.g.* on p. 41. The description of the pre-reduction, reduction, and post-reduction phases of the development of the sex cells is so condensed as to be hardly accurate. It was Farmer and Moore, not Moore and Walker, who introduced the terms pre-maiotic, maiotic, and post-maiotic, which they spell as here, and not as the author spells them on p. 17. These criticisms notwithstanding, it is a pleasure to welcome a new edition which embodies the recent work on human embryology, and the present volume will be of great value to workers in this subject.

### MISCELLANEOUS ITEMS.

The Theory of Ions : A Consideration of Its Place in Biology and Therapeutics. By William Tibbles, Hon. M.D.Chicago, LL.D., L.R.C.P.E., M.R.C.S., L.S.A., etc. (London : Rebman, Limited. Pp. viii. + 131. Price, 2s. 6d. net.)

There is a growing school which believes that a direct evolution from non-living to living matter will some day be proved. Studies upon substances in solutions, upon radio-activity in its various kinds, upon biological chemistry, and so forth, have led to an increased knowledge of the physical state of the particles of molecules which are undergoing dissociation. The dissociated particles are termed ions. They may be atoms, but they are more often groups of atoms, or parts of atoms, in a state of rapid movement, and charged with electricity. The dissociation of combined elements and the formation of ions or unattached atoms having an electrical reaction is brought about by various influences, the best known of which are heat, light, chemical action, electricity, Röntgen and other rays. The book before us is a more or less popular exposition of the behaviour of these ions, and their significance as far as is at present known; and the underlying stratum upon which this exposition is built is the idea that there has been an evolution not only of more complex living bodies out of simpler, nor merely of more complex non-living bodies out of simpler, but also of simple living from complex non-living groups of ions. The author's suggestion appears to be that the phenomena of life will some day be explained, and that the lines along which researches upon the question are most likely to be fruitful are those which are given by the study of these ions.

Britain's Blot. By J. F. Sutherland, M.D. (Edin.), F.R.S.E. (William Green and Sons. Price, 3s. net.)

The chief objection to this book is its title, which is misleading. With such a sensational heading one is scarcely prepared for the sound and impartial treatment of the problem. Dr. Sutherland, with a wealth of statistics, endeavours to secure a hearing for some sound reform in dealing with criminals. "Rescidivism" he uses as a term which describes two classes of habitual offender, the active evil-doer and the passive "waster." The author, though offering nothing very new, is certainly to be admired for his thorough grasp of the problem and the practical suggestions he makes. We are not very much impressed by his diagrams of concentric circles. In conclusion, it is interesting to notice that he is totally opposed to all drastic and Draconian methods in dealing with this class of criminal. Environment, he thinks, is of probably the greater importance in the majority of cases.

The Black Death.—Messrs. Bell and Sons have recently published this book, by Dr. Gasquet, a contribution to plague literature—and at the same time to mediæval medicine—which will be welcomed by the student of medicine. Dr. Gasquet begins with the introduction of the plague into Europe in the spring of 1348, along the line of the caravan routes of Asia Minor, and traces the swift march of the disease across Europe to our Western Islands. Perhaps we have not sufficiently realised the great influence of epidemics of infectious disease upon Early English life, but the reader of these fascinating pages cannot fail to appreciate this influence. In addition to the careful collection of well-known records such as "Decameron," the author also gives us many interesting side-lights from ecclesiastical sources not so readily accessible to the average reader; for example, the Patent Rolls and the "Inquisitiones Post Mortem." Moreover Dr. Gasquet's clear skilful treatment of the subject gives the book a style of its own, and brings out forcibly the widespread effects of the Black Death on literature, education, architecture—indeed, on the entire social order of mediæval England.

The Solar System. By Charles Lane Poor, Professor of Astronomy in Columbia University. (London: John Murray, 1908. Price 6s. net.)

Since physicians ceased to consider astrology as an integral part of the medical curriculum, medicine and the study of the solar system have had few points of direct contact. Perhaps the only problem in medicine on which the sun's satellites have any bearing is that of the menstrual cycle; but this is a complicated question with which Professor Poor's volume naturally does not deal, as it is intended for popular edification. His account of the solar system is well worth reading as a preliminary to the serious adoption of astronomy as a recreation, and as such it can be recommended to the medical profession.

On Means for the Prolongation of Life. By Sir Hermann Weber, M.D., F.R.C.P. (London : Bale, Sons and Danielsson. Third Edition. Price 4s. 6d. net.)

Sir H. Weber's admirable sermon is preached from the text that work and moderation are the main sources of health, happiness, and long life, and a very helpful and practical discourse it is. Incidentally it may be observed that the prolongation of life implies the preservation of health, and as a sane and reasonable outline of the principles of personal hygiene this book is worthy of attention both from the medical profession and the public at large. In this new edition the most important additions are those pages dealing with the common articles of diet, their digestibility, and their *rôle* in the nutrition of the body; these add perceptibly to the usefulness of this interesting monograph.

Health Resorts of Europe. By T. Linn, M.D. (London : The Health Resorts Bureau. Fifteenth Edition, 1908. Price 2s. 6d. net.)

In this volume is presented an enormous mass of information as to the numerous resorts of Europe frequented for climatic or other reasons by those seeking to preserve or to regain health. By careful condensation and classification this information is rendered readily available for reference, but it is necessarily, for reasons of space, not very full. The medical information is, we suppose, based upon the claims of the physicians who practise in the various localities. We notice that one French resort is held out as suitable for "scrofulous children with ganglionic and osseous manifestations of that diathesis," a hard saying which sounds euphonious but conveys—to us at least—remarkably little. In old-established manuals such as this to keep the lists of doctors, hotels, and so on up to date is no doubt difficult; we notice one or two instances in which this has not been done, though probably they are the exception and not the rule. As a handy reference-book we recommend this publication to the medical profession.

